# A case of recurrent vaccine-triggered Gianotti–Crosti syndrome

**DOI:** 10.2144/fsoa-2020-0200

**Published:** 2021-03-04

**Authors:** Joy Assaf, Farid Stephan, P Zeinaty, Roland Tomb

**Affiliations:** 1 Department of Dermatology, Hôtel-Dieu de France University Hospital, Saint-Joseph University, Beirut, Lebanon

**Keywords:** acrodermatitis, child, Gianotti–Crosti syndrome, recurrence, trigger, vaccination

## Abstract

Gianotti–Crosti syndrome (GCS) is a self-limited benign dermatosis, clinically characterized by a monomorphic papular or papulovesicular eruption symmetrically distributed on the limbs and face of children. Various viral and vaccine triggers have been associated with GCS. Recurrences are uncommon but have been reported. We report a case of recurrent vaccine-triggered GCS.

## Lay abstract

The aim of this paper is to give pediatricians and dermatologists a more precise idea of recurrent Gianotti–Crosti syndrome that occurs following vaccine administration to eventually prevent unnecessary investigations in future patients or vaccination restrictions.

Gianotti–Crosti syndrome (GCS), also known as papular acrodermatitis of childhood is clinically characterized by an acute onset of a papular or papulovesicular monomorphic eruption distributed symmetrically on the extremities, buttocks and face while sparing the torso, palms and soles [1,2]. Various viral, bacterial and vaccine triggers have been described in association with GCS with Epstein–Barr virus and hepatitis B virus as the two most common pathogens [1,3]. GCS is usually diagnosed clinically and supportive treatment is required. We report the case of a 2-year-old child who had two episodes of vaccine associated GCS over 3 months.

## Case report

**Figure 1.**
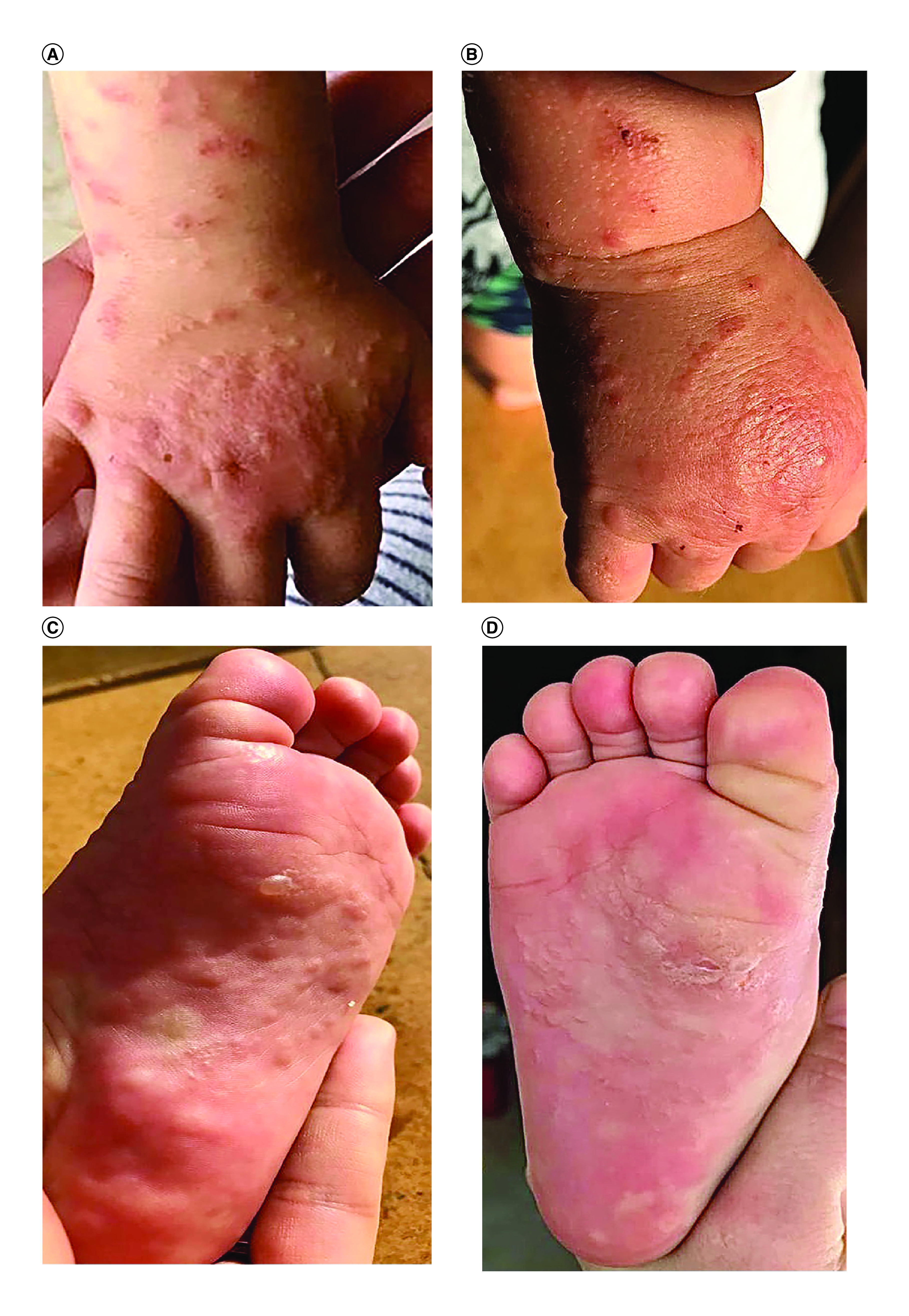
Clinical features of the patient on initial presentation (A–D). Erythematous monomorphic excoriated papules on the right arm and hand **(A–B).** Vescico-bullous transformation on the soles **(C)** followed by postbullous eruption **(D).**

**Figure 2.**
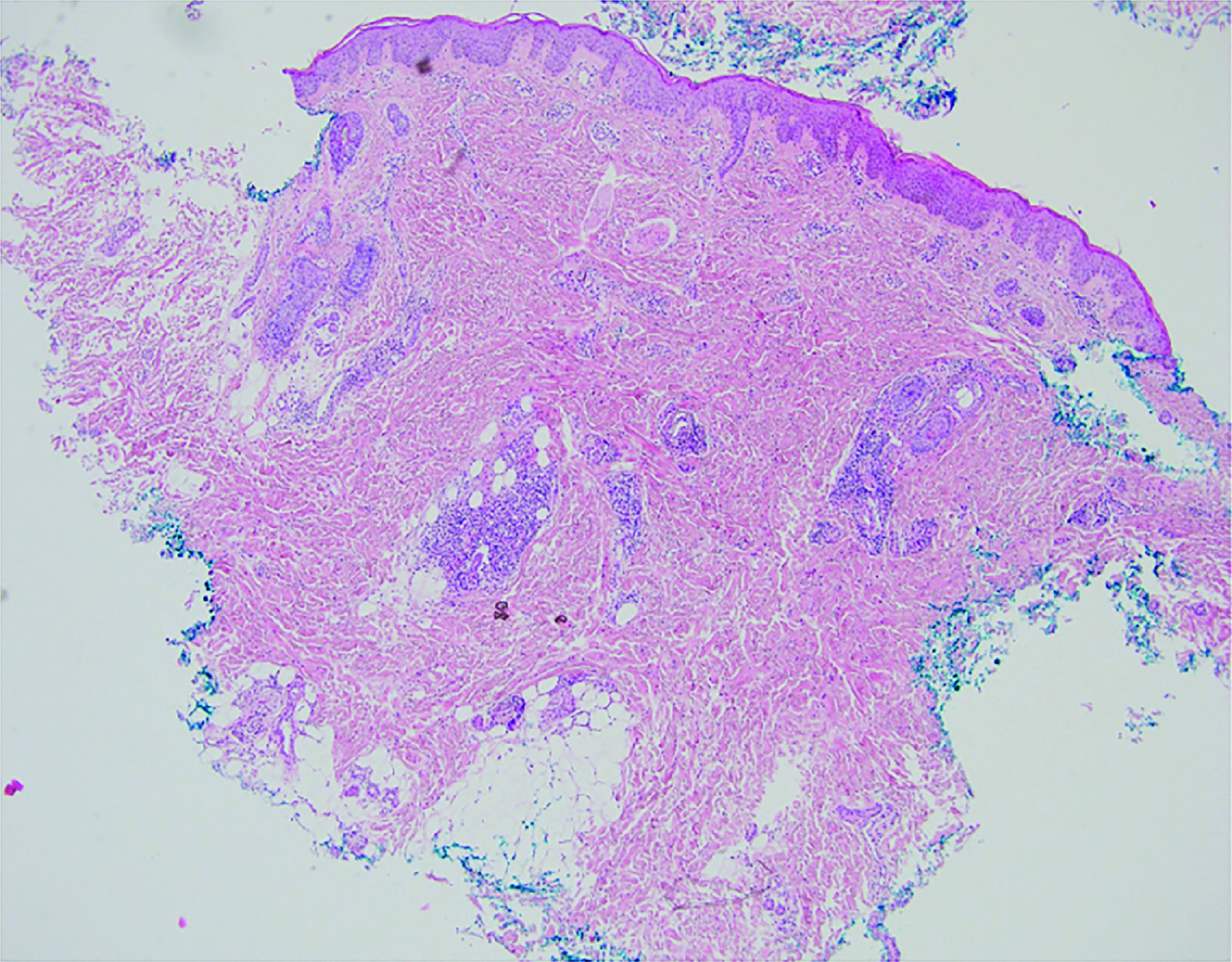
Histopathological features of biopsy specimen stained for hematoxylin-eosin showing mild acanthosis and a moderately intense dermal lympho-histiocytic infiltrate (5x magnification).

A previously healthy 2-year-old girl presented with a 4-week history of a cutaneous pruritic eruption that initially appeared on her legs and subsequently progressed to affect her soles, arms and face sparing the trunk and palms. The child had already been treated three-times with topical administration of benzyl-benzoate lotion as a trial treatment for scabies without any improvement. History revealed that 2 weeks prior to the eruption, the child had received hepatitis B vaccination. On physical examination, she was afebrile with erythematous papules and vesicles on the cheeks, arms, hands and legs (Figure 1A & B) with bullous transformation on the soles (Figure 1C & D). There was no lymphadenopathy or hepatosplenomegaly. The patient experienced intense pruritus causing sleep disturbances. In the view of parents’ demand and atypical clinical presentation, a skin biopsy was performed and histopathologic examination of a specimen from the left buttock revealed the presence of parakeratosis and mild acanthosis in the epidermis. Focally, mild interface dermatitis is noted without apoptosis. Moderately intense perivascular and periadnexial lympho-histiocytic infiltrate is also noted in the superficial and mid-dermis with focal exocytosis of lymphoid cells (Figure 2). The child was diagnosed with atypical GCS following hepatitis B vaccination. Because symptoms were unresponsive to topical betamethasone 0.05% and oral antihistaminic drug, she received oral administration of dexamethasone 0.5 mg/5 ml for 4 days and the eruption resolved partially.

**Figure 3.**
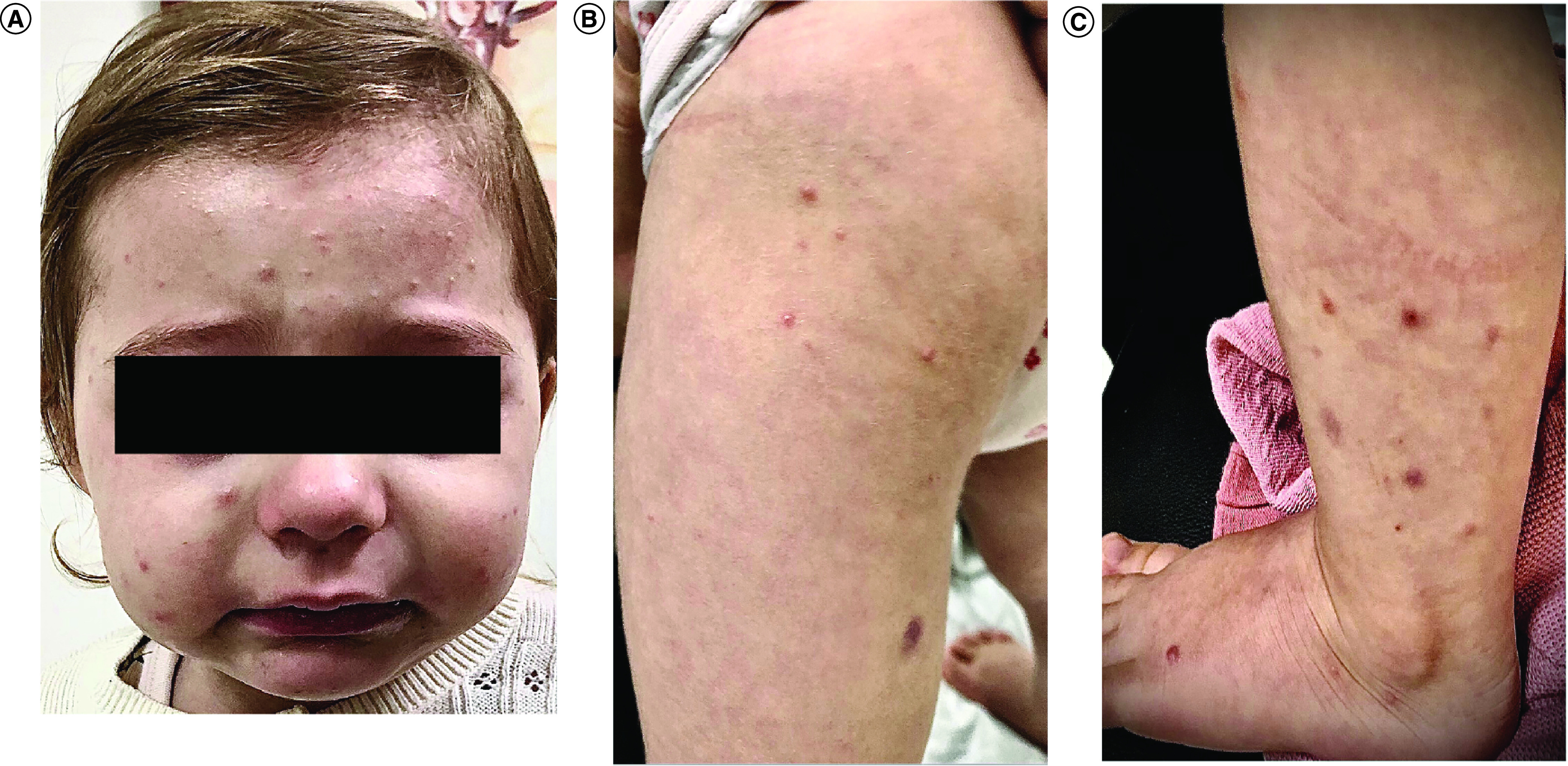
Clinical features of the patient on relapse. **(A)** Erythematous monomorphic papules on the face. **(B & C)** Papulovesicular eruption on the extremities.

Three months later, the child presented with a similar papulovesicular monomorphic pruritic eruption localized on the face, arms and legs, sparing the trunk as well (Figure 3A–C). The MMR vaccine (measles, mumps and rubella) was administered 7 days prior to the eruption. A history of intercurrent viral illness was noted by the parents at the time of vaccination. The patient was treated only with topical corticosteroids and an oral antihistaminic drug.

## Discussion

Gianotti–Crosti syndrome is a self-limited dermatosis first described by Crosti and Gianotti in 1957 [1]. It is a relatively common condition worldwide, but frequently remains underdiagnosed. It appears most commonly in infants between 1 and 6 years old, however similar cases were described in adults [1,4]. Studies showed that GCS occurred more frequently during spring and summer and in patients with personal or family history of atopy [5,6]. The exact pathogenesis of GCS remains uncertain; however, two main hypotheses were reported; the first one was an IgE mediated response supported by the association of GCS with elevated serum IgE and atopy. The second one was a local delayed (type 4) hypersensitivity reaction in the dermis to various pathogens and vaccines [1,7]. While initial studies showed that hepatitis B virus was the most common causative pathogen, many other agents have since been reported including Epstein–Barr virus, cytomegalovirus, influenza virus, hepatitis C and hepatitis A virus. Similarly, bacteria such as *Bartonella henselae*, *Borrelia burgdorferi* and other agents have been described as possible causes of GCS [8]. Vaccine triggers have also been associated with GCS such as oral polio vaccine, measles, mumps and rubella vaccine, influenza vaccine, hemophilus influenza type b vaccine, hepatitis B and hepatitis A vaccine [3,4,9].

The condition is clinically characterized by a sudden onset of a papular or papulovesicular monomorphic eruption distributed symmetrically. Individual lesions are usually flesh-colored to red-brown, flat-topped and occasionally scaly found predominantly on the extensor surfaces of the extremities, buttocks and face [1]. The trunk, palms and soles are usually spared, but, if affected, diagnosis should not be ruled out [5]. Systemic manifestations are possible including mild fever, arthralgia, generalized lymphadenopathy, diarrhea and hepatosplenomegaly [2,5]. Pruritus can also be noted [4]. Spontaneous resolution will usually occur in 10 days to 6 months; however, various reports noted that eruption can last as long as 12 months.

GCS is usually clinically diagnosed without the need of laboratory tests. Peripheral blood may show leukocytosis or leucopenia, depending on the inciting event. Elevated liver enzymes are usually seen in patients with Epstein–Barr virus, cytomegalovirus or hepatitis virus infection [1,5]. In case of atypical presentation or continuous eruption for more than 6 months, a skin biopsy is required to rule out other diagnosis [1]. Histopathologic findings are nonspecific and include mild focal spongiosis, edema of the papillary dermis with mild acanthosis, hyperkeratosis, parakeratosis with superficial perivascular lymphohistiocytic inflammatory infiltrate in the upper dermis and dilated dermal capillaries. Immunohistochemical analysis shows that the majority of lymphocytes are CD4^+^ and CD8^+^ T cells [5,7]. Since GCS is a benign and self-limited condition, parents should be reassured and advised that treatment is usually not needed [5].

Our patient experienced atypical GCS associated with hepatitis B vaccination, and 3 months later a recurrence of classic GCS associated with MMR vaccination. A relapse of Gianotti–Crosti syndrome has only been described three-times in the literature. One report noted recurrence with two different viral triggers: anicteric hepatitis B infection and 5 months later a recurrence associated with rubella infection [10]. While the second report documented recurrence of vaccine-associated GCS following intramuscular influenza vaccination [3]. Also, Lam reported two episodes of GCS following the same H1N1 vaccine administered 2 weeks apart [11]. Furthermore, only two reports described a single episode of GCS associated with MMR vaccination with no recurrences noted [12,13].

The pathogenesis of vaccine induced GCS is uncertain. One study reported that within the lesions, the inflammatory infiltrate consists of dendritic cells and T cells suggesting that GCS is characterized by a vaccination and/or a virus-induced hypersensitivity immune response [14,15]. In our patient, GCS occurred primarily after hepatitis B vaccination with a relapse following MMR vaccination which supports previous reports suggesting that vaccination stimulates immune induction. However, also in our case, there was an active viral infection reported by the parents at the time of MMR immunization prior to the second episode of GCS. We admit that this intercurrent viral illness casts doubt as to whether or not the second episode was truly triggered by the vaccination or whether the association with the vaccination was co-incidental.

Moreover, previous studies reported an atypical clinical presentation in GCS not associated with hepatitis B, characterized by a polymorphic eruption of erythematous or purpuric papules, vesicles and blisters asymmetrically distributed, frequently coalescing forming large edematous and pruritic lesions [16,17]. Admittedly, this was consistent with our case. In fact, the child presented multiple erythematous papules and vesicles with bullous transformation on the soles, severe pruritus and chronic evolution over several months.

Thus, our patient had atypical clinical presentation with pathology compatible with GCS and responded relatively well to systemic and topical steroids with the exception of occasional mild flares. This confirms the benign character of this syndrome. In the rising of vaccine hesitancy and refusal of vaccination by parents, dermatologists should stress the importance of the favorable prognosis of this self-limited condition [1]. Therefore, future vaccinations are not contraindicated.

## Conclusion & future perspective

In summary, this report describes an atypical GCS-like reaction following the hepatitis B vaccination and a typical GCS eruption following MMR vaccination with the possible trigger of intercurrent viral infection. Although in the literature only few cases of recurrent GCS are reported, in our clinical experience this is not a rare phenomenon. Probably, recurrent GCS is more likely underreported rather than really rare. Thus, there is a need for analysis of more case reports of recurrent vaccine-induced GCS in order to identify the mechanism of the onset and the etiopathogenesis behind this entity and to determine the tight association between GCS and vaccination.

**Executive summary**

- Gianotti–Crosti syndrome is a self-limited benign dermatosis associated with multiple viral and vaccine triggers.
- Recurrences are uncommon but have been scarcely reported in the literature.
- This case presentation confirms the benign character of this syndrome and suggests that Gianotti–Crosti syndrome is characterized by a vaccination induced immune response and/or a virus-induced hypersensitivity immune response.
- Pediatricians and dermatologists should stress the importance of the favorable prognosis of this self-limited condition in order to prevent unnecessary investigations in future patients or vaccination restriction.

## References

[1] Leung AKC, Sergi CM, Lam JM, Leong KF. Gianotti–Crosti syndrome (papular acrodermatitis of childhood) in the era of a viral recrudescence and vaccine opposition. World J. Pediatr. 15(6), 521–527 (2019). 31134587 10.1007/s12519-019-00269-9

[2] Pedreira RL, Leal JM, Silvestre KJ, Lisboa AP, Gripp AC. Gianotti–Crosti syndrome: a case report of a teenager. An. Bras. Dermatol. 91(1 Suppl. 5), 163–165 (2016). 28300930 10.1590/abd1806-4841.20164410 PMC5325029

[3] Metelitsa AI, Fiorillo L. Recurrent Gianotti–Crosti syndrome. J. Am. Acad. Dermatol. 65(4), 876–877 (2011). 21920247 10.1016/j.jaad.2010.08.008

[4] Al Dhaheri HS, Al Kaabi A, Kara Hamo Y, Al Kaabi A, Al Kaabi S, Al Tatari H. Unusual presentation of Gianotti–Crosti syndrome due to Epstein–Barr virus infection. Case Rep. Dermatol. Med. 2016, 1017524 (2016). 28050291 10.1155/2016/1017524 PMC5165146

[5] Brandt O, Abeck D, Gianotti R, Burgdorf W. Gianotti–Crosti syndrome. J. Am. Acad. Dermatol. 54(1), 136–145 (2006). 16384769 10.1016/j.jaad.2005.09.033

[6] Ricci G, Patrizi A, Neri I, Specchia F, Tosti G, Masi M. Gianotti–Crosti syndrome and allergic background. Acta Derm. Venereol. 83(3), 202–205 (2003). 12816156 10.1080/00015550310007210

[7] Chuh AA. Diagnostic criteria for Gianotti–Crosti syndrome: a prospective case–control study for validity assessment. Cutis. 68(3), 207–213 (2001). 11579787

[8] Drago F, Javor S, Ciccarese G, Parodi A. Gianotti–Crosti syndrome as presenting sign of cytomegalovirus infection: a case report and a critical appraisal of its possible cytomegalovirus etiology. J. Clin. Virol. 78, 120–122 (2016). 27017141 10.1016/j.jcv.2016.03.009

[9] Shibata T, Yanagishita T, Oshima Y, Watanabe D. Case of Gianotti–Crosti syndrome following varicella zoster virus vaccination. J. Dermatol. 46(1), e36–e38 (2019). 29897138 10.1111/1346-8138.14500

[10] Patrizi A, Di Lernia V, Neri I, Ricci G. An unusual case of recurrent Gianotti–Crosti syndrome. Pediatr. Dermatol. 11(3), 283–284 (1994). 10.1111/j.1525-1470.1994.tb00611.x 7971574

[11] Lam JM. Atypical Gianotti–Crosti syndrome following administration of the AS03-adjuvanted H1N1 vaccine. J. Am. Acad. Dermatol. 65(4), e127–e128 (2011). 21920234 10.1016/j.jaad.2011.04.005

[12] Velangi SS, Tidman MJ. Gianotti–Crosti syndrome after measles, mumps and rubella vaccination. Br. J. Dermatol. 139(6), 1122–1123 (1998). 9990393 10.1046/j.1365-2133.1998.2576j.x

[13] Lacour M, Harms M. Gianotti-Crosti syndrome as a result of vaccination and Epstein–Barr virus infection. Eur. J. Pediatr. 154(8), 688–689 (1995). 10.1007/BF02079084 7588978

[14] Magyarlaki M, Drobnitsch I, Schneider I. Papular acrodermatitis of childhood (Gianotti–Crosti Disease). Pediatr. Dermatol. 8(3), 224–227 (1991). 1745633 10.1111/j.1525-1470.1991.tb00865.x

[15] Baldari U, Monti A, Righini MG. An epidemic of infantile papular acrodermatitis (Gianotti–Crosti syndrome) due to Epstein–Barr virus. Dermatol. Basel Switz. 188(3), 203–204 (1994). 10.1159/000247139 8186509

[16] Marcassi AP, Piazza CA de D, Seize MB de MP Atypical Gianotti–Crosti syndrome. An. Bras. Dermatol. 93(2), 265–267 (2018). 29723358 10.1590/abd1806-4841.20186726 PMC5916403

[17] Endo M, Mori H, Morishima T. On infantile papular acrodermatitis (Gianotti disease) and infantile papular-similvesicular acrodermatitis (Gianotti syndrome). J. Dermatol. 2(1), 5–14 (1975). 1104683 10.1111/j.1346-8138.1975.tb00934.x

